# Bereaved Family Members' Perceived Care at the End of Life for Patients with Noncancerous Respiratory Diseases

**DOI:** 10.1089/pmr.2021.0034

**Published:** 2021-10-06

**Authors:** Ryosuke Imai, Atsushi Mizuno, Mitsunori Miyashita, Kohei Okafuji, Atsushi Kitamura, Yutaka Tomishima, Torahiko Jinta, Naoki Nishimura, Tomohide Tamura

**Affiliations:** ^1^ Department of Pulmonary Medicine, Thoracic Center, St. Luke's International Hospital, Tokyo, Japan.; ^2^ Cardio Vascular Center, St. Luke's International Hospital, Tokyo, Japan.; ^3^ Division of Palliative Nursing, Health Sciences, Tohoku University Graduate School of Medicine, Sendai, Japan.

**Keywords:** caregiver issues, dementia, end-stage respiratory failure

## Abstract

***Background:*** Data regarding the quality of end-of-life care for patients with noncancerous illnesses are lacking.

***Objective:*** This study aimed to evaluate end-of-life care for patients with noncancerous respiratory disease from the perspective of bereaved family members and explore the factors associated with the quality of patient death and care.

***Design:*** This cross-sectional study included patients who had died of noncancerous respiratory disease in general wards of pulmonary department in Japan between 2014 and 2016 and conducted an anonymous self-report questionnaire survey for the patients' bereaved family members.

***Measurements:*** We evaluated overall satisfaction with care and the quality of death and end-of-life care using the Good Death Inventory (GDI) and Care Evaluation Scale (CES), respectively. A multiple linear regression analysis was performed to explore the factors associated with these outcomes.

***Results:*** In total, 130 questionnaires were distributed, and the effective response rate was 38% and 50 patients were included (median age: 82 [range 58–101] years; 37 men [74%]). Primary diagnoses at death included 29 cases of pneumonia (58%), 15 interstitial lung disease (30%), and 3 chronic obstructive pulmonary disease (6%). Of the bereaved family members, 26 (52%) were spouses, and 19 (38%) were children (median age [range]: 68 [33–102] years, 15 men [30%]). The overall CES and GDI scores (mean ± standard deviation) were 77 ± 15 and 79 ± 15, respectively. The presence of dementia was an independent factor associated with high CES and GDI scores in the multiple linear regression analysis.

***Conclusions:*** In patients who died of noncancerous respiratory disease, the presence of dementia could be associated with the higher quality of patient death and care. In dementia, an understanding of the terminal nature of this condition may lead to an appropriate end-of-life care.

## Introduction

One of the most important goals of palliative care is the achievement of a “good death” or “good dying process.”^1^ Despite advanced investigation into the quality of end-of-life care for cancer patients, limited data are available for patients with noncancerous illnesses.

High-quality end-of-life care entails relieving and minimizing the physical, psychosocial, and spiritual suffering of patients and their families, and assessing the quality of the end-of-life care consists mainly of three factors: structure, process, and outcome.^2^ In palliative care, it is difficult to evaluate end-of-life care through patients directly because patients who are dying usually have functional decline and a variety of burden of disease, such as dyspnea, delirium, anxiety, noisy respiratory secretions, worsening pain, and nausea. Therefore, end-of-life care evaluation from a multifaceted perspective, including these three factors, using surrogate evaluation of bereaved family members has become a global standard.^3–5^

In Japan, a nationwide survey of bereaved family members^6,7^ has been conducted to examine the quality of end-of-life care in cancer patients, using the Care Evaluation Scale (CES),^8^ which was developed to evaluate palliative care structure and processes, and the Good Death Inventory (GDI),^9^ which was developed to measure the quality of life in cancer patients.

Moreover, noncancerous respiratory diseases often develop into respiratory failure at the terminal phase, and the physical and mental burden on patients and their family members is considered large; therefore, active palliative care is necessary. However, limited data are available regarding the quality of end-of-life care in patients with noncancerous respiratory disease.

The purpose of this study was to evaluate end-of-life care for noncancerous respiratory patients from the perspective of bereaved family members and explore the factors associated with the quality of patient death and care. We hypothesized that family satisfaction among patients who are expected to be difficult to care for would be low, such as those on ventilators, on opioids, and with dementia.

## Materials and Methods

### Participants and procedure

This single-center retrospective cross-sectional study conducted an anonymous self-report questionnaire survey at St. Luke's International Hospital in Tokyo, Japan. Patients who had died of noncancerous respiratory disease in general wards of pulmonary department between January 1, 2014 and December 31, 2016 were included in the study. Inclusion criteria were (1) patient whose death cause was considered a noncancerous respiratory disease by the attending physician; (2) patient died in general wards; (3) patient was ≥20 years old; and (4) bereaved family member was ≥20 years old. We excluded individuals whose addresses could not be identified and those who would have suffered serious psychological distress with participation, as determined by the primary physician and nurse.

The questionnaire was mailed to eligible bereaved family members. Participants returned the completed questionnaire to the secretariat office within one month. We sent a reminder to nonresponders one month after mailing the questionnaire.

The study was approved by the Institutional Review Board (No. 17-R165). By completing the questionnaire all the participants were considered to have consented to participate in the study because it contained a statement regarding consent.

### Data collection related to patients

Medical records were reviewed for each decedent retrospectively to obtain demographic information, underlying diseases, primary diagnosis at death, cause of death, the presence of do-not-resuscitate (DNR) orders, use of oxygen devices, medical treatment, opioid use, and clinical outcomes. Underlying diseases were determined based on the primary physician's description in the death report.

### Data collection related to bereaved family members

We asked the bereaved family members for the following information: age, gender, relationship with the patient, health status during admission, frequency of attending to the patient, and presence of other caregivers. Overall care satisfaction and the structure and process of care (CES) and achievement of good death (GDI) were used to evaluate the quality of end-of-life care.

### CES short version

We used the short version of the CES to evaluate care quality. The CES was developed to measure end-of-life care from the perspective of bereaved family members, focusing on the structure and process of care.^8^ The original version of the CES comprises 10 domains (help with decision making for patient, help with decision making for family, physical care by physician, physical care by nurse, psychoexistential care, environment, cost, availability, coordination of care, and family burden) with 28 attributes. The questionnaire was designed to allow respondents to evaluate the structure and process of end-of-life care by rating the need for improvement for each item on a 6-point Likert scale (1 = improvement is highly necessary, 2 = improvement is quite necessary, 3 = improvement is necessary, 4 = improvement is somewhat necessary, 5 = improvement is slightly necessary, and 6 = improvement is not necessary). The total score was transformed to a scale ranging from 0 to 100, with higher scores indicating better care. The short version of the CES consists of 10 items representing each domain, and the validity and reliability of the scale have been confirmed.

### GDI short version

We used the short version of the GDI^9^ to evaluate the quality of death. The GDI was developed to measure the quality of the patient's death from the bereaved family members' perspective. Attributes were generated based on previous qualitative and quantitative studies.^1,10^ The original version of the GDI consists of 18 domains, with 10 core and 8 optional domains, with a total of 54 attributes. The 10 core domains evaluate attributes that most Japanese people have consistently rated as important, and the eight optional domains evaluate attributes that have not been consistently rated as important and depend on individual values. The short version of the GDI consists of 18 representative items from each domain, and the validity and reliability of the scale have been confirmed. We asked participants to evaluate each attribute using a 7-point Likert scale (1 = absolutely disagree, 2 = disagree, 3 = somewhat disagree, 4 = unsure, 5 = somewhat agree, 6 = agree, and 7 = absolutely agree). Total scores were calculated by summing scores for all attributes, with higher scores indicating the achievement of a good death.

### Statistical analysis

Data are presented as median (range) or means ± standard deviations (SDs) for continuous variables as appropriate and frequencies (percentages) for categorical variables. The continuous variables representing the background factors were bisected, in principle, close to the median and at clinically relevant values. The relationship between CES scores, GDI scores, and background factors were then analyzed. Differences between groups were analyzed using a Student's *t* test or analysis of variance as appropriate. We then performed a multiple regression analysis using the step-down method for variables with *p* < 0.10 in univariate analysis and a known related factor, patients' age. All statistical tests were two tailed, and the level of significance was set at *p* < 0.05. All analyses were performed using R software, version 3.5.2 (R Foundation for Statistical Computing).

## Results

**FIG. 1.**
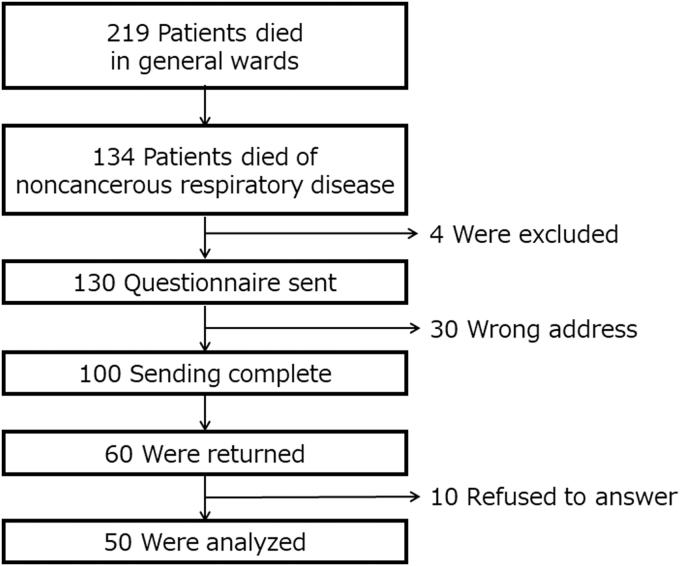
Outline of the study.

In total, 134 patients met the inclusion criteria. Four patients were excluded. Of the 130 questionnaires mailed to patients' bereaved family members, 30 were undeliverable and 60 were returned (Fig. 1). Of the returned questionnaires, 10 participants refused to answer the questionnaires; therefore, 50 questionnaires were analyzed (effective response rate 38%). The patients' characteristics are shown in Table 1. The patients' median age (range) was 82 (58–101) years, and 37 were men (74%). Regarding comorbidity, 22 (44%), 11 (22%), 8 (16%), and 7 (14%) had been diagnosed with heart disease, diabetes mellitus, dementia, and chronic kidney disease, respectively. Three (6%) had malignancies (breast, pancreatic, and ureteral cancers); however, at the time of death, the attending physician determined that the cause of death was not the cancer.

**Table 1.** Patients' Demographic and Clinical Characteristics

Characteristics	*N* = 50
Age (years)	82 (58–101)
Men	37 (74)
Primary diagnosis at death	
Pneumonia	29 (58)
Interstitial lung disease	15 (30)
Tuberculosis	3 (6)
Chronic obstructive pulmonary disease	3 (6)
Cause of death	
Respiratory failure	27 (54)
Hypotension	10 (20)
Carbon dioxide narcosis	8 (16)
Other	5 (10)
Underlying disease	
Heart disease	22 (44)
Diabetes mellitus	11 (22)
Dementia	8 (16)
Chronic kidney disease	7 (14)
Malignancy	3 (6)
Religion	
Buddhism	21 (42)
Atheism	19 (38)
Christianity	4 (8)
Shinto^a^	1 (2)
Other	5 (10)
Admitted at first visit	14 (28)
DNR at admission	29 (58)
Time from DNR to death (days)	8 (0–316)
Respiratory failure (days)	7 (3–16)
Duration of hospital stay (days)	13 (1–339)
Opioid use	16 (32)
Oxygen device at death	
None	3 (6)
Conventional oxygen	26 (52)
High-flow nasal cannula	13 (26)
Noninvasive ventilation	6 (12)
Mechanical ventilation	2 (4)

Continuous variables are expressed as median (range), and categorical data are expressed as *n* (%).

^a^ Japanese traditional religion.

DNR, do-not-resuscitate

Primary diagnoses at death included 29 cases of pneumonia (58%), 15 of interstitial lung disease (30%), and 3 of chronic obstructive pulmonary disease (6%). Twenty-nine patients (58%) had requested DNR orders at admission, and 42 (84%) died without using ventilation. Sixteen (32%) had used opioids, and more than half of patients died of respiratory failure. The bereaved family members' characteristics are presented in Table 2. Of the bereaved family members, 26 (52%) were spouses, and 19 (38%) were children (median age [range]: 68 [33–102] years, 15 men [30%]).

**Table 2.** Bereaved Family Members' Demographic and Clinical Characteristics

Characteristics	*N* = 50
Age (years)	68 (33–102)
Men	15 (30)
Relationship with the deceased	
Spouse	26 (52)
Child	19 (38)
Other	5 (10)
Health status during admission	
Good	12 (24)
Moderate	29 (58)
Fair	7 (14)
Bad	2 (4)
Frequency of patient attendance	
Everyday	30 (60)
4–6 Days/week	7 (14)
3 Days or less/week	13 (26)
Presence of other caregivers	36 (72)

Continuous variables are expressed as median (range) or means ± SDs for continuous variables as appropriate, and categorical data are expressed as *n* (%).

SD, standard deviation.

### Comparison of CES and GDI scores according to patients' characteristics

The overall CES and GDI scores (mean ± SD) were 77 ± 15 and 79 ± 15, respectively. The relationship between indicators of end-of-life care (CES and GDI scores) assessed by the bereaved family members and the patients' characteristics are presented in Table 3. The factors significantly associated with high CES and GDI scores were the presence of dementia and family members who could change patients' attendance or nursing care during their final hospital stays. Having a DNR order at admission, the duration of respiratory failure, opioid use, using ventilator at time of death, and cause of death were not correlated with CES and GDI scores. In the multiple linear regression analysis, the presence of dementia was an independent factor affecting high CES and GDI scores (regression coefficient: 13.4, 95% confidence interval [CI]: 2.68–24.1, *p* = 0.0155, regression coefficient: 15.4, 95% CI: 4.04–26.7, *p* = 0.00894, respectively; Table 4) The responses to the items on CES and GDI were compared between patients with dementia and those without (Supplementary Appendix). Of the items on the CES, the bereaved family members of dementia patients were highly satisfied with the following: “Physicians, nurses, and staff endeavored so that the patient's hope would be accomplished” and “There is good cooperation among staff members such as physicians and nurses.” Regarding the items on the GDI, they were satisfied with the following: “Physical and psychological comfort,” “Maintaining hope and pleasure,” and “Environmental comfort.”

**Table 3.** Relationship between Indicators of End-of-Life Care Assessed by the Bereaved Family Members and the Characteristics of Patients and Bereaved Family Members

Patients' characteristics	CES	*p* ^a^	GDI	*p* ^a^
Overall	77 ± 15	—	79 ± 15	—
Age (years)				
<85 (*n* = 24)	76 ± 15	0.404	76 ± 19	0.643
≥85 (*n* = 26)	79 ± 15		78 ± 17	
Gender				
Male (*n* = 37)	77 ± 14	0.634	76 ± 19	0.445
Female (*n* = 13)	79 ± 17		80 ± 16	
Primary diagnosis at death				
Pneumonia (*n* = 29)	75 ± 14	0.466	74 ± 19	0.439
Interstitial lung disease (*n* = 15)	81 ± 17		79 ± 16	
Other (*n* = 6)	80 ± 15		88 ± 17	
Cause of death				
Respiratory failure (*n* = 27)	78 ± 16	0.787	77 ± 20	0.771
Hypotension (*n* = 10)	80 ± 18		79 ± 14	
Carbon dioxide narcosis (*n* = 8)	75 ± 18		77 ± 16	
Other (*n* = 5)	73 ± 18		68 ± 20	
Underlying disease				
Heart disease				
Yes (*n* = 22)	78 ± 14	0.795	81 ± 17	0.196
No (*n* = 28)	77 ± 16		74 ± 18	
Diabetes mellitus				
Yes (*n* = 11)	84 ± 7	0.102	81 ± 12	0.423
No (*n* = 39)	76 ± 16		76 ± 19	
Dementia				
Yes (*n* = 8)	88 ± 9	0.0222	91 ± 13	0.0164
No (*n* = 42)	76 ± 15		74 ± 15	
CKD				
Yes (*n* = 7)	76 ± 12	0.788	74 ± 15	0.615
No (*n* = 43)	78 ± 15		77 ± 19	
Religion				
Yes (*n* = 31)	77 ± 16	0.864	76 ± 21	0.698
No (*n* = 19)	78 ± 14		78 ± 13	
DNR at admission				
Yes (*n* = 29)	76 ± 14	0.381	76 ± 20	0.792
No (*n* = 21)	80 ± 16		78 ± 16	
Time from DNR to death				
<7 Days (*n* = 22)	78 ± 16	0.985	75 ± 21	0.557
≥7 Days (*n* = 28)	78 ± 15		78 ± 15	
Duration of respiratory failure				
<7 Days (*n* = 25)	75 ± 15	0.392	74 ± 19	0.349
≥7 Days (*n* = 25)	79 ± 15		79 ± 17	
Duration of hospital stay				
<14 Days (*n* = 22)	77 ± 15	0.819	76 ± 22	0.748
≥14 Days (*n* = 28)	78 ± 14		78 ± 13	
Opioid use				
Yes (*n* = 16)	79 ± 15	0.583	79 ± 15	0.541
No (*n* = 34)	77 ± 15		76 ± 19	
Ventilation at death				
Yes (*n* = 8)	76 ± 10	0.681	76 ± 15	0.942
No (*n* = 42)	78 ± 16		77 ± 19	
Bereaved family members' characteristics				
Age (years)				
<70 (*n* = 28)	76 ± 16	0.370	75 ± 16	0.559
≥70 (*n* = 22)	80 ± 14		78 ± 21	
Gender				
Male (*n* = 15)	78 ± 14	0.830	77 ± 20	0.826
Female (*n* = 35)	77 ± 16		75 ± 18	
Relationship to the deceased				
Spouse (*n* = 26)	80 ± 14	0.572	77 ± 18	0.982
Child (*n* = 19)	75 ± 17		77 ± 19	
Other (*n* = 5)	77 ± 13		76 ± 18	
Health status during admission				
Good (*n* = 12)	78 ± 15	0.675	83 ± 13	0.403
Moderate (*n* = 29)	77 ± 16		77 ± 14	
Fair (*n* = 7)	76 ± 14		83 ± 22	
Bad (*n* = 2)	90 ± 14		68 ± 1	
Frequency of patient attendance				
Every day (*n* = 30)	77 ± 13	0.988	79 ± 13	0.502
4–6 Days/week (*n* = 7)	78 ± 19		75 ± 18	
≤3 Days/week (*n* = 13)	77 ± 18		72 ± 28	
Presence of other caregivers				
Yes (*n* = 36)	80 ± 14	0.0585	80 ± 16	0.208
No (*n* = 14)	71 ± 17		74 ± 14	

Continuous variables are expressed as means ± SDs.

^a^ Comparison scores of each attribute using Student's *t* test or ANOVA test.

ANOVA, analysis of variance; CES, Care Evaluation Scale; CKD, chronic kidney disease; DNR, do not resuscitate, GDI, Good Death Inventory.

**Table 4.** Multiple Linear Regression Analysis for Attributes of High Care Evaluation Scale and Good Death Inventory Scores

CES model	Regression coefficient	95% CI	*p*
Patient's age ≥85	4.61	−3.28 to 7.12	0.246
Dementia	13.4	2.68 to 24.1	0.0155
Presence of other caregivers	8.36	−0.35 to 17.1	0.0594
GDI model			
Patient's age ≥85	2.22	−6.25 to 10.6	0.595
Dementia	15.4	4.04 to 26.7	0.00894

CI, confidence interval.

## Discussion

The results of this study demonstrated that the presence of dementia tended to be associated with higher quality of death and care in patents with noncancer respiratory illnesses. In contrast, the duration of respiratory failure, using a ventilator at the time of death, or cause of death were not correlated with the quality of patient death and care.

In a Japanese nationwide survey, which used the CES and GDI to examine the quality of care and death for cancer patients, the mean CES scores for patients who had died in designated cancer centers and palliative care units were 68 ± 21 and 78 ± 17, respectively, whereas mean GDI scores were 78 ± 17 and 85 ± 15, respectively.^7^ The scores for cancer patients in palliative care units were higher compared with those observed in this study; however, a direct comparison is not possible because of differences in patients' characteristics. The Japanese Ministry of Health, Labor and Welfare requires that palliative care units in Japan assign higher numbers of nurses per patient, relative to other wards; allow larger floor space in patient rooms for each patient, relative to other wards; and include a patient-only kitchen, family waiting room, and a lounge on the ward, which could have contributed to high levels of satisfaction in bereaved family members in the nationwide survey. In a previous study examining the factors contributing to family satisfaction with inpatient palliative care in cancer patients, Morita et al. performed a cross-sectional survey through mail, using the Satisfaction scale for Family Members Receiving Inpatient Palliative Care and identified the following significant determinants of family satisfaction: the number of nurses at night, the presence of attending medical social workers, patient age, family age, floor space per bed, duration of admission, and extra charges for private rooms.^3^ In addition, Takeuchi et al. conducted a survey through mail to examine CES and GDI scores for 9684 bereaved family members of decedents at 103 palliative care units in Japan and showed that significant determinants of these scores in the evaluation of care were as follows: private room rate, independent facilities, palliative care physician on night duty, the number of nurses present at night, sending letters to every bereaved family, holding memorial services for every bereaved family, and having a religious background.^11^ It is necessary to actively utilize palliative care units in future and consider organization-related factors and care for bereaved family members, even for patients with noncancerous illnesses.

Based on the results of this study, it is hypothesized that the presence of dementia could be associated with higher quality of patient death and care. Dementia is a terminal illness, with poor prognosis after the onset of complications such as infection or eating problems.^12^ In addition, advanced dementia can cause pain, dyspnea, and anorexia; however, managing pain toward the end of life is extremely poor. This is because patients with dementia have difficulties in expressing their symptoms and instead exhibit behavioral changes such as agitation, distress, social withdrawal, or resistive behavior, leading to underdetected or undertreated pain.^13^ However, using the appropriate assessments and treatment for pain has been shown to improve the quality of life of patients with dementia.^14^ In this study, the bereaved family members were highly satisfied with the following items, especially regarding end-of-life care: “Physicians, nurses, and staff endeavored so that the patient's hope would be accomplished,” “Physical and psychological comfort,” and “Environmental comfort” (Supplementary Appendix). These results suggest the importance of assessing and caring for the dementia patients' distress and pain, which is difficult to assess.

In contrast, the severity of respiratory failure at death and cause of death was not correlated with the quality of patient death and care. Mularski et al. conducted surveys of family members of intensive care unit decedents using the Quality of Dying and Death instrument and reported that higher scores were associated with the management of pain and events.^5^ However, these scores were not related to APACHE II scores, the use of a mechanical ventilator, or kidney dialysis, which is consistent with the current findings.

The study had some limitations. First, it was a single-center retrospective study with a small sample size. Moreover, the generalizability of these results may be limited since the quality of end-of-life care is affected by race, religion, and environment. Further research is required to verify our findings in larger populations. In addition, the response rate was 38%, and response bias could have occurred. However, we do not consider this a fatal flaw of the study, because the response rate was higher than the average for public surveys in Japan. Second, recall bias is a major issue in self-report questionnaire surveys. The family satisfaction of the bereaved family members of those who died more recently and that of the family members of those who died a long time ago may differ. Therefore, in this study, we decided to minimize the effect of recall bias by limiting the participants to those who had lost a loved one within three years. Third, the GDI also included inappropriate items that made it difficult to determine whether or not patients with dementia felt, such as “Not being a burden to others.” As a result, the GDI score may have tended to be high. Moreover, the CES and GDI were originally developed to assess the quality of end-of-life care for patients with cancer and has not been validated for use in noncancerous patients or non-DNR cases. However, the CES evaluates the structure and process of care, including physical and psychoexistential care, help with decision making for patients or family, environment, and cost. In addition, the GDI comprises domains that most Japanese people consider necessary for a good death, these are thought to be common to both cancer and noncancer patients. Therefore, we believe that these scales can be used for noncancerous patients. Future studies should further investigate what is good death in patients with dementia.

## Conclusions

In conclusion, the results indicated that the presence of dementia was associated with the higher quality of death and care in patients who had died of noncancerous respiratory disease. In the case of dementia, an understanding of the terminal nature of this condition may lead to appropriate end-of-life care.

## Supplementary Material

Supplemental data

## Abbreviations Used

CES: Care Evaluation Scale
DNR: do-not-resuscitate
GDI: Good Death Inventory
SD: standard deviation
