# Supplementary material for: Bereaved Family Members' Perceived Care at the End of Life for Patients with Noncancerous Respiratory Diseases
Source: Palliat Med Rep. 2021 Oct 6;2(1):265–71. doi: 10.1089/pmr.2021.0034 (PMC8675229; doi:10.1089/pmr.2021.0034)
Supplement: Supplemental data [file Supp_DataSA1.docx]

Supplementary Appendix: Items on CES and GDI compared between patients with dementia and those without

| Items | Dementia | No dementia | *P* value* |
| --- | --- | --- | --- |
| CES |  |  |  |
| Physicians endeavored to relieve physical discomfort of the patient | 5.4 ± 0.7 | 4.6 ± 1.3 | 0.098 |
| Nurses have adequate knowledge and skills to alleviate physical symptoms of the patient | 5.4 ± 0.5 | 4.8 ± 1.3 | 0.178 |
| Physicians have adequate knowledge and skills to alleviate physical symptoms of the patient | 5.5 ± 0.8 | 4.9 ± 1.0 | 0.121 |
| Physicians, nurses, and staff endeavored so that the patient’s hope would be accomplished | 5.6 ± 0.5 | 4.7 ± 1.2 | 0.049 |
| Physician gave sufficient explanation to the family about condition and the medical treatment | 4.9 ± 1.7 | 4.0 ± 1.7 | 0.171 |
| Hospital or room was convenient and comfortable | 5.5 ± 0.5 | 4.6 ± 1.3 | 0.061 |
| Consideration was given to the health of family | 4.8 ± 0.9 | 4.0 ± 1.4 | 0.154 |
| The total cost is reasonable | 5.0 ± 0.7 | 4.0 ± 1.5 | 0.071 |
| Admission (use) is possible, when necessary, without waiting | 5.5 ± 0.5 | 5.2 ± 1.1 | 0.430 |
| There is good cooperation among staff members such as physicians and nurses | 5.6 ± 0.5 | 4.6 ± 1.3 | 0.033 |
| GDI |  |  |  |
| Physical and psychological comfort | 5.9 ± 1.1 | 4.5 ± 1.6 | 0.021 |
| Dying in a favorite place | 4.9 ± 2.0 | 4.2 ± 1.6 | 0.316 |
| Maintaining hope and pleasure | 5.1 ± 1.8 | 3.6 ± 1.6 | 0.017 |
| Good relationship with medical staff | 6.1 ± 1.1 | 5.1 ± 1.5 | 0.065 |
| Not being a burden to others | 5.1 ± 2.0 | 4.0 ± 1.6 | 0.071 |
| Good relationship with family | 5.0 ± 1.9 | 4.9 ± 1.5 | 0.839 |
| Independence | 3.0 ± 2.0 | 2.9 ± 2.0 | 0.807 |
| Environmental comfort | 6.1 ± 1.1 | 4.9 ± 1.2 | 0.008 |
| Being respected as an individual | 6.3 ± 1.2 | 5.9 ± 0.8 | 0.279 |
| Life completion | 5.7 ± 1.4 | 4.7 ± 1.7 | 0.121 |

Continuous variables are expressed as means ± standard deviations. CES = Care Evaluation Scale, GDI = Good Death Inventory. *Comparison scores of each attribute using Student’s *t* test.
